# Antioxidant capacity and peptidomic analysis of in vitro digested *Camelina sativa L. Crantz* and *Cynara cardunculus* co-products

**DOI:** 10.1038/s41598-024-64989-3

**Published:** 2024-06-24

**Authors:** Davide Lanzoni, Francesca Grassi Scalvini, Elena Petrosillo, Simona Nonnis, Gabriella Tedeschi, Giovanni Savoini, Arianna Buccioni, Guido Invernizzi, Antonella Baldi, Carlotta Giromini

**Affiliations:** 1 Department of Veterinary Medicine and Animal Sciences (DIVAS), Università degli Studi di Milano, Via Dell’Università 6, 29600 Lodi, Italy; 2 CRC, Innovation for Well-Being and Environment, Università degli Studi di Milano, 20122 Milano, Italy; 3 Dipartimento di Scienze e Tecnologie Agrarie, Alimentari, Ambientali e Forestali, University of Florence, Piazzale delle Cascine 18, 50144 Firenze, Italy; 4 Centro Interdipartimentale di Ricerca e Valorizzazione Degli Alimenti, University of Florence, viale Pieraccini 6, 50139 Firenze, Italy; 5 Institute for Food, Nutrition and Health, University of Reading, Reading, RG6 5EU UK

**Keywords:** Antioxidant activity, Bioactive peptides, Camelina, Cardoon, NanoLC-MS/MS, Peptidomics, Proteomic analysis, Peptide delivery

## Abstract

In recent decades, the food system has been faced with the significant problem of increasing food waste. Therefore, the feed industry, supported by scientific research, is attempting to valorise the use of discarded biomass as co-products for the livestock sector, in line with EU objectives. In parallel, the search for functional products that can ensure animal health and performances is a common fundamental goal for both animal husbandry and feeding. In this context, camelina cake (CAMC), cardoon cake (CC) and cardoon meal (CM), due valuable nutritional profile, represent prospective alternatives. Therefore, the aim of this work was to investigate the antioxidant activity of CAMC, CC and CM following in vitro digestion using 2,2′-azinobis-(3-ethylbenzothiazoline-6-sulphonic acid) (ABTS), Ferric reducing antioxidant power (FRAP) and oxygen radical absorbance capacity (ORAC) assays. Total phenolic content (TPC) and angiotensin converting enzyme (ACE) inhibitory activity, actively involved in modulating antioxidant properties, were also studied. Further, a peptidomic analysis was adopted to substantiate the presence of bioactive peptides after in vitro digestion. The results obtained confirmed an interesting nutritional profile of CAMC, CC and CM and relevant antioxidant and ACE inhibitory activities. In particular, considering antioxidant profile, CM and CC revealed a significantly higher (10969.80 ± 18.93 mg TE/100 g and 10451.40 ± 149.17 mg TE/100 g, respectively; *p* < 0.05) ABTS value than CAMC (9511.18 ± 315.29 mg TE/100 g); a trend also confirmed with the FRAP assay (306.74 ± 5.68 mg FeSO_4_/100 g; 272.84 ± 11.02 mg FeSO_4_/100 g; 103.84 ± 3.27 mg FeSO_4_/100 g, for CC, CM and CAMC, respectively). Similar results were obtained for TPC, demonstrating the involvement of phenols in modulating antioxidant activity. Finally, CAMC was found to have a higher ACE inhibitory activity (40.34 ± 10.11%) than the other matrices. Furthermore, potentially bioactive peptides associated with ACE inhibitory, anti-hypertensive, anti-cancer, antimicrobial, antiviral, antithrombotic, DPP-IV inhibitory and PEP-inhibitory activities were identified in CAMC. This profile was broader than that of CC and CM. The presence of such peptides corroborates the antioxidant and ACE profile of the sample. Although the data obtained report the important antioxidant profile of CAMC, CC, and CM and support their possible use, future investigations, particularly in vivo trials will be critical to evaluate and further investigate their effects on the health and performance of farm animals.

## Introduction

In recent decades, the food system has been faced with the significant problem of increasing food waste. Indeed, as reported by the Food and Agriculture Organisation of the United Nations (FAO), approximately 1.3 billion tonnes of food are lost or wasted globally each year^1^. For this reason, the European Union has implemented multiple strategies to ensure the sustainability of the food and feed sector. In this context, the Agenda 2030, later supported by the Green Deal, plays a major role, whose goal is to ensure a competitive, clean and circular economy^2,3^.

For this reason, scientific research is investigating the use of non-edible biomass produced along the food chain as co-products (any product obtained from different agro-industrial processes) for livestock^4^. As shown by Govoni et al.^5^, the introduction of co-products (11–16%) to replace energy-rich food crops (such as cereals) would conserve 15.4–27.8 Mha of land, 3–19.6 km^3^ of blue water and 74.2–137.8 km^3^ of green water, representing an important strategy to ensure environmental sustainability. To date, although many co-products have already been tested (pomace, beet pulp, hemp seed cakes), new ones need to be investigated, meeting EU objectives^6–8^.

As reported by Turco^9^ and Singh et al.^10^, the co-products of camelina (*Camelina Sativa* L. Crantz) and cardoon (*Cynara cardunculus*) are attracting the interest of scientific research thanks to their low environmental impact and interesting nutritional profile. More specifically, camelina and cardoon seeds are characterised by a protein content of 25.9% and 16.7% on dry matter (DM) and by a lipid percentage of 38.9 ± 1.26% and 25–30% on DM, respectively^9–11^. As described by Singh et al.^10^ and Petropoulos et al.^12^, the fatty acid profile of camelina and cardoon seeds, rich in polyunsaturated (55.6% and 65.43 ± 0.08%, respectively) and low in saturated fatty acids (9.04% and 13.23 ± 0.07%, respectively), prompted the food and feed industry to isolate the lipid fraction for nutritional and nutraceutical purposes, creating in parallel scrap products. The main scrap matrices of this processing are definitely, camelina cake (CAMC), cardoon cake (CC) and cardoon meal (CM), which, due to their important nutritional profile, can find application in animal nutrition, as reported by Giromini et al.^13^, Lolli et al.^14^ and Serrapica et al.^15^. However, the characterisation of the functional profile is still at an early stage. As reported by Corino and Rossi^16^, investigating the medical/functional characteristics of plant matrices plays a key role in the feed and livestock sector. Among the most important, the antioxidant profile is certainly one of the main players involved in protecting welfare and ensuring high animal performance.

Therefore, in the light of the above, the aim of this work was to investigate the functional characteristics, particularly the antioxidant profile, of CAMC, CC and CM during the in vitro digestion process, with 2,2′-azinobis-(3-ethylbenzothiazoline-6-sulphonic acid) (ABTS), ferric reducing antioxidant power (FRAP) and oxygen radical absorbance capacity (ORAC) assays. At the same time, total phenolic content (TPC) and angiotensin converting enzyme (ACE) inhibitory activity, parameters actively involved in modulating the antioxidant profile, were investigated. Finally, the peptidomic approach was adopted to identify the main bioactive peptides resulting from the digestive process.

## Results

### Determination of nutritional profile and digestibility

The nutritional profile is shown in Table 1.

**Table 1:** Nutritional profile of CAMC, CC and CM.

Sample	DM	ASHES	CP	FATS	NDF	ADF	ADL
CAMC	92.08 ± 0.16^a^	4.76 ± 0.43^a^	31.42 ± 0.41^a^	7.86 ± 0.19^a^	67.40 ± 0.04^a^	32.88 ± 0.53^a^	15.50 ± 0.49^a^
CC	93.23 ± 0.27^b^	4.49 ± 0.48^a^	16.88 ± 0.98^b^	6.61 ± 0.09^b^	50.81 ± 2.67^b^	37.00 ± 0.44^b^	13.13 ± 0.65^a^
CM	93.70 ± 0.19^b^	6.11 ± 0.25^a^	16.58 ± 0.84^b^	0.53 ± 0.05^c^	67.12 ± 0.55^a^	33.76 ± 2.29^a^	12.05 ± 0.93^a^

Nutritional profile of camelina cake (CAMC), cardoon cake (CC) and cardoon meal (CM) (% w/w on DM basis). Data are presented as mean ± standard error of mean (SEM). (n = 3).

*DM* dry matter, *CP* crude protein, *NDF* neutral detergent fibre, *ADF* acid detergent fibre, *ADL* acid detergent lignin.

Different superscript letters in columns indicate statistically significant differences (*p* < *0.05*).

As reported in Table 1, CAMC, CC and CM are characterised by an interesting nutritional profile. More specifically, although ashes were highly comparable, this trend was not observed for DM content. In fact, the DM recorded for CAMC (92.08 ± 0.16%) showed statistically significant differences (*p* < 0.05) compared to CC (93.23 ± 0.27%) and CM (93.70 ± 0.19%). In parallel, CAMC (31.42 ± 0.41%) presented a significantly higher crude protein (CP) content (*p* < *0.05*) than CC (16.88 ± 0.98%) and CM (16.58 ± 0.84%). The same trend was also confirmed for lipid concentration (7.86 ± 0.19, 6.61 ± 0.09, 0.53 ± 0.05%, for CAMC, CC and CM, respectively). As shown in Table 1, the fibrous fractions reported similar values. More precisely, CAMC showed no difference for neutral detergent fibre (NDF), acid detergent fibre (ADF) and acid detergent lignin (ADL) compared to CM. Although CC had a significantly lower NDF content than other matrices, this was not observed for both ADF (37.00 ± 0.44%) with statistically higher values (*p* < 0.05) than CAMC (32.88 ± 0.53%) and CM (33.76 ± 2.29%), and ADL (13.13 ± 0.65%) with a highly comparable content compared to CAMC and CM (15.50 ± 0.49% and 12.05 ± 0.93%, respectively).

**Figure 1:**
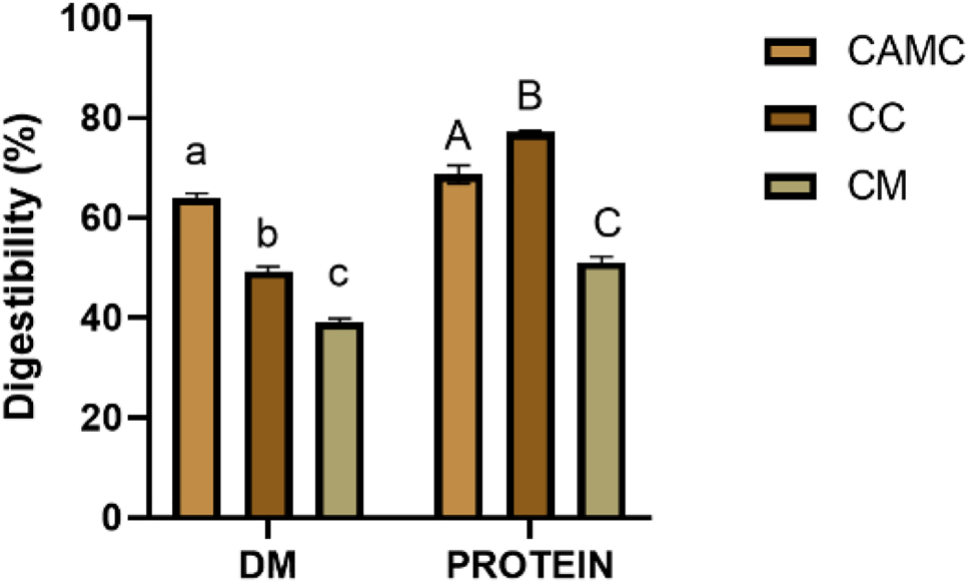
Dry matter (DM) and protein digestibility of camelina cake (CAMC), cardoon cake (CC) and cardoon meal (CM). Values in % are presented as mean ± standard error of mean (SEM) (n = 3). Small letters indicate statistically significant differences for DM digestibility, capital letters for protein digestibility (*p* < 0.05).

At the same time, as can be noted in Fig. 1, CAMC, CC and CM are also characterised by an interesting DM/protein digestibility.

CAMC (64.08 ± 0.77%) had a significantly higher (*p* < 0.05) DM digestibility than CC (49.30 ± 0.96%) and CM (39.14 ± 0.74%), with also significant differences between the latter two. However, this trend was not observed for the protein one. More specifically, in this case, CC (77.24 ± 0.24%) showed significantly higher digestibility (*p* < 0.05) than CAMC and CC (68.69 ± 1.83, 51.24 ± 0.93%, respectively).

### Total phenolic content and antioxidant activity

**Figure 2:**
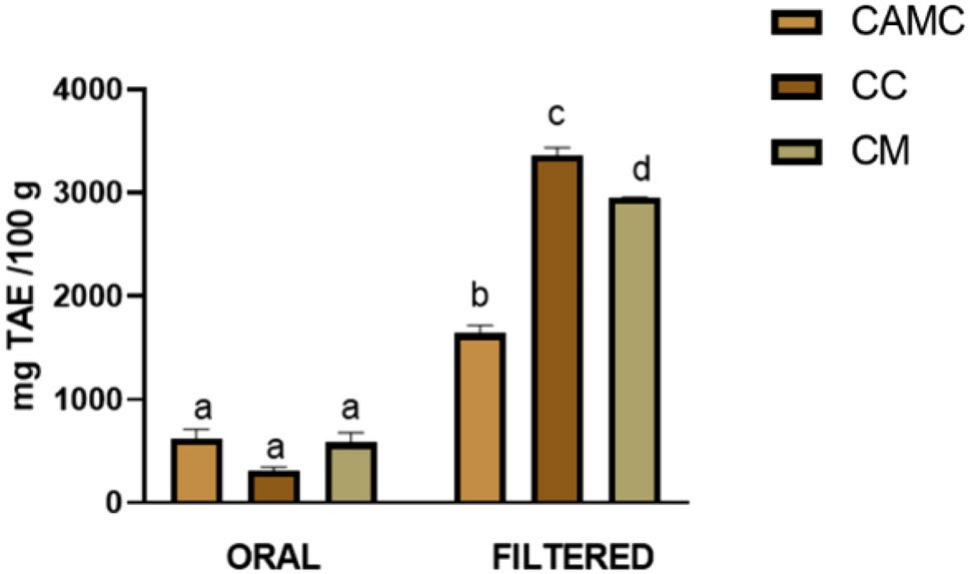
Total Phenolic Content of camelina cake (CAMC), cardoon cake (CC) and cardoon meal (CM). Data are presented as mean ± standard error of mean (SEM), (n = 3). Different superscript letters in columns indicate significant different data (*p* < 0.05).

Figure 2 shows the TPC of CAMC, CC, CM at the end of the oral phase and following filtration (10 kDa).

As reported, at the end of the oral phase, the samples had similar values (621.19 ± 88.98 mg TAE/100 g; 304.13 ± 42.43 mg TAE/100 g; 581.64 ± 95.15 mg TAE/100 g, for CAMC, CC and CM, respectively). In parallel, filtration resulted in a significantly higher TPC than that just described, with significant differences (*p* < 0.05) between each matrix analysed (1636.67 ± 78.89 mg TAE/100 g; 3357.60 ± 79.82 mg TAE/100 g; 2947.98 ± 8.15 mg TAE/100 g, for CAMC, CC and CM, accordingly).

**Figure 3:**
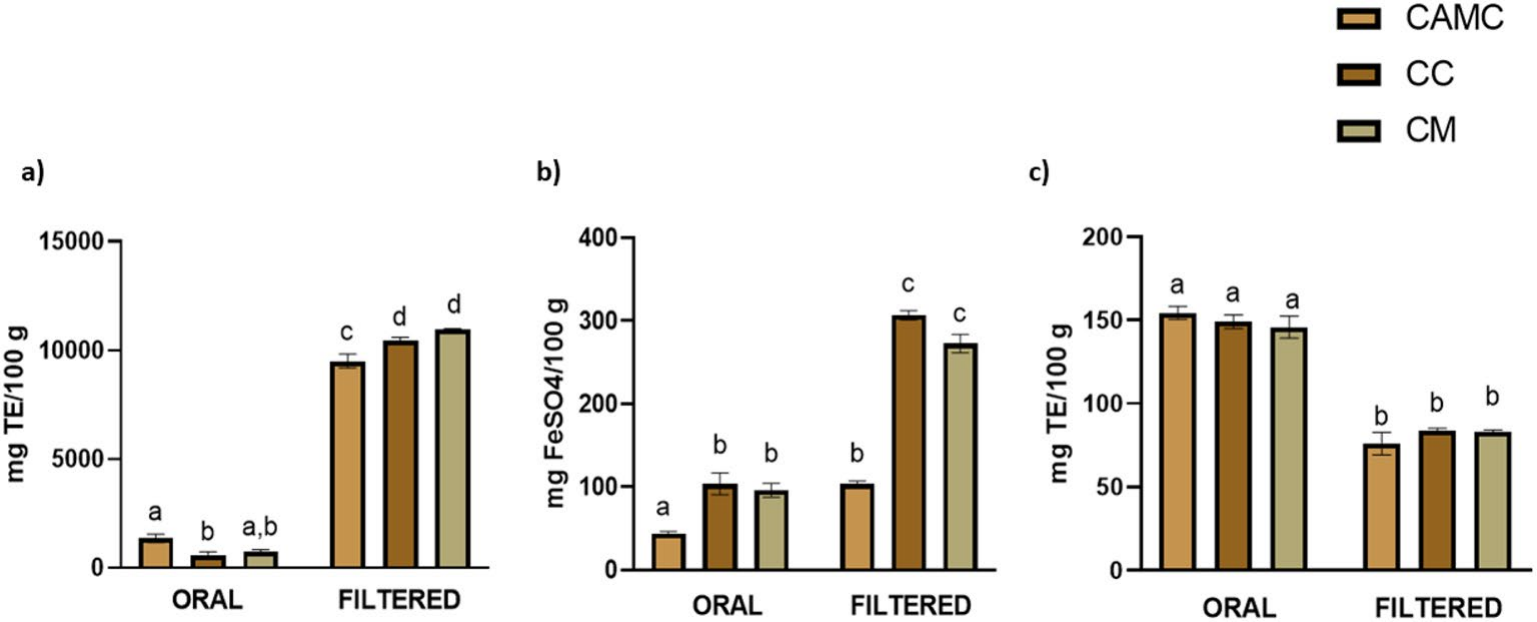
Antioxidant activity of camelina cake (CAMC), cardoon cake (CC) and cardoon meal (CM). Data are presented as mean ± standard error of mean (SEM), (n = 3). Different superscript letters in columns indicate significant different data (*p* < 0.05). (**a**) ABTS assay; (**b**) FRAP assay; (**c**) ORAC assay.

At the same time, as shown in Fig. 3, CAMC, CC and CM are also characterised by high antioxidant activity.

As can be noted, ABTS and FRAP trends correlated with that of TPC. More specifically, for the ABTS assay, although at the end of the oral phase, CAMC (1354.38 ± 189.30 mg TE/100 g) showed significantly greater antioxidant activity (*p* < 0.05) than CC (590.83 ± 154.46 mg TE/100 g) and comparable to CM (764.64 ± 135.32 mg TE/100 g), this was not observed after filtration, with significant (*p* < 0.05) lower values (9511.18 ± 315.29 mg TE/100 g) than the similar ones of CC (10,451.40 ± 149.17 mg TE/100 g) and CM (10,969.80 ± 18.93 mg TE/100 g). Even with the FRAP method, better results were obtained for CC and CM (103.78 ± 12.98 mg FeSO_4_/100 g; 96.10 ± 8.42 mg FeSO_4_/100 g, respectively) with significant differences (*p* < 0.05) compared to CAMC (44.10 ± 2.54 mg FeSO_4_/100 g) at the end of the oral phase. This trend was also observed following the filtration phase (306.74 ± 5.68 mg FeSO_4_/100 g; 272.84 ± 11.02 mg FeSO_4_/100 g; 103.84 ± 3.27 mg FeSO_4_/100 g, for CC, CM and CAMC, respectively).

However, as shown in Fig. 3, ORAC presented an opposite trend to that observed for ABTS and FRAP, with significantly higher values (*p* < 0.05) for the oral phase (154.49 ± 3.91 mg TE/100 g; 149.09 ± 4.23 mg TE/100 g; 145.79 ± 6.60 mg TE/100 g, for CAMC, CC and CM, respectively) than those observed following filtration (75.99 ± 6.82 mg TE/100 g; 83.96 ± 1.24 mg TE/100 g; 83.29 ± 0.90 mg TE/100 g for CAMC, CC and CM, respectively), with no statistically significant difference between the matrices at any stage of the digestive process.

### ACE inhibitory activity

Interesting results were observed in the ACE inhibition assay. The highest inhibitory effect of ACE occurred in CAMC sample (40.34 ± 11.11%) after in vitro digestion, which was significantly higher (*p* < 0.05) compared to the effect observed before digestion. This trend, although not statistically significant, was reported for CC, with an ACE inhibitor rate of 10.88 ± 9.05% following the digestive process. No effect were detected in CM at the end of intestinal phase.

### CAMC, CC and CM peptidomic profile

**Figure 4:**
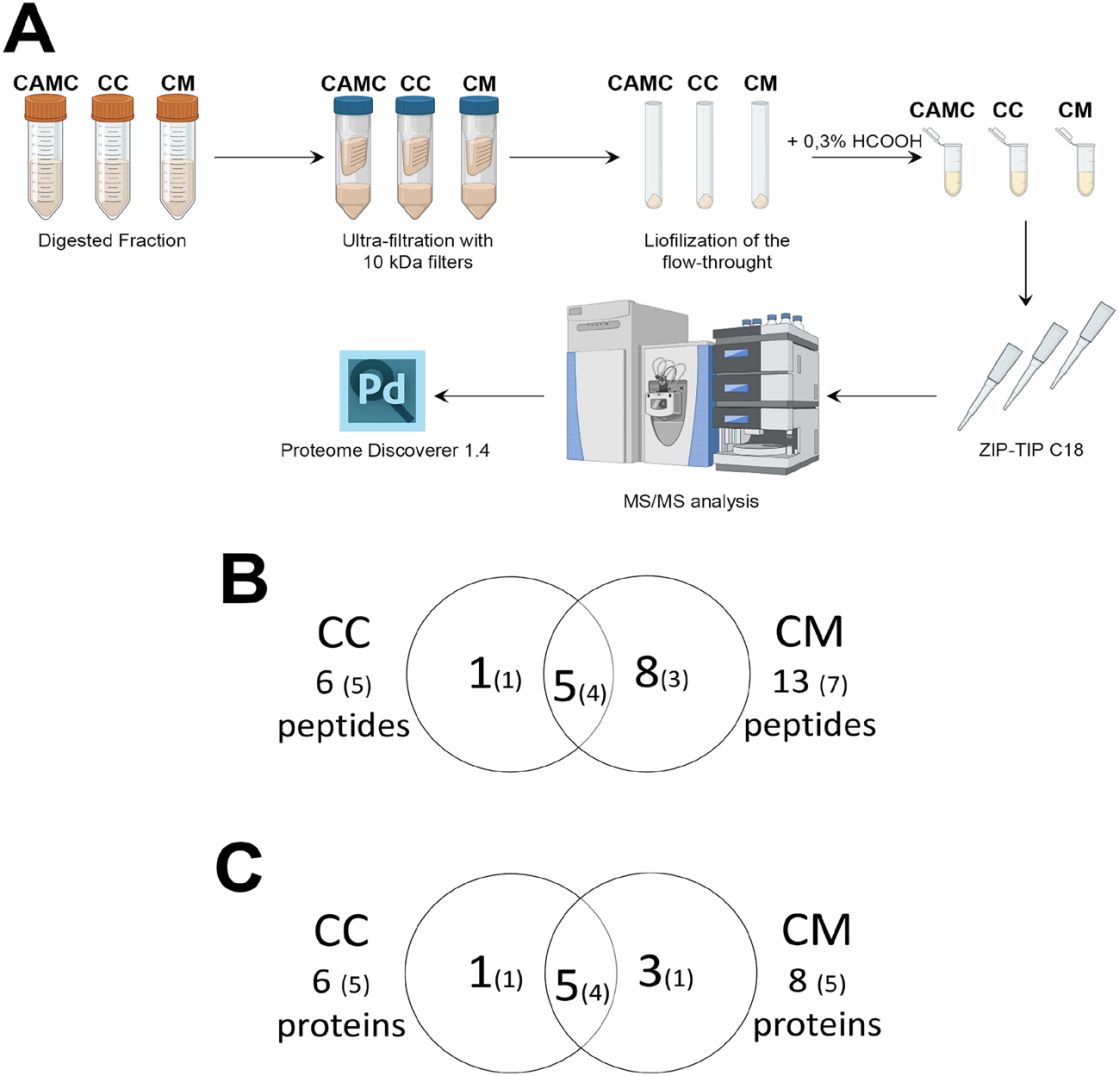
Schematic representation of the shotgun label-free peptidomic analysis. (**A**) Overview of the workflow applied on CAMC, CC and CM specimens. (**B**) Venn diagram of all peptides identified from the comparison CC vs CM. Unique peptides are indicated in brackets. (**C**) Venn diagram of the proteins that generated all peptides from the comparison CC vs CM. Proteins that generated unique peptides are indicated in brackets.

The peptidomic analysis was carried out as schematized in Fig. 4A and allowed to identify all the peptides present in CAMC, as well as, those that were exclusively present or were common between CC and CM samples. In particular, 382 peptides were identified in CAMC (Supplementary Table S1), whose 188 are unique peptides and are produced by the proteolysis of 180 proteins. Well in accordance with the lower protein content described above in cardoon, less peptides were identified in CC and CM (6 and 13 respectively), of which 5 and 7 are unique peptides (Supplementary Table S2). A specific comparison identifies 5 peptides common in CC and CM samples (4 are unique peptides) and 1 and 8 exclusively present in CC and CM, respectively (Fig. 4B). All these peptides were generated from 6 and 8 proteins in CC and CM (Fig. 4C), respectively, of which 5 common to all samples: clpP-like protease, 30S ribosomal protein S3, photosystem II Q(b) protein (D1), NADH-plastoquinone oxidoreductase subunit 2 and the hypothetical chloroplast RF21 protein. In parallel, the search for potential bioactive peptides resulted in the identification of 30 peptides in CAMC, as shown in Table 2 (SATPdb database) and 3 (DFBP database), and one in CM (SATPdb and DFBP databases), as reported in Table 4.

**Table 2:** List of potentially bioactive peptides found in CAMC specimens in SATPdb database.

CAMC SATPdb					
Bioactivity	Peptides identified	Database SATPdb sequence	Protein origin	Gene	Protein name
Ace Inhibitors (7)	EFCGGTDTKR**VIKP**TEDKRFEEmTnTVGMIEHYMnInHWVC	VIKP	1109068111	LOC104746755	Eukaryotic translation initiation factor 3 subunit C
	GA**GAGP**GLGGGIGPDNTLVFFmHDILGGSNP	GAGP	727457383	LOC104786832	Dirigent protein 10-like
	GQDHVQPSNMDSPAKKqKTSSqGPDVqIDSGEETLRNPSmE**RPYL**C	RPYL	1109077495	LOC104750981	Probable inactive tRNA-specific adenosine deaminase-like protein 3
	GQGGqLLSPYqGSYNqGQGTPLPGQGQE	YQGS	727647923	LOC104771657	Multiple organellar RNA editing factor 4, mitochondrial-like
	IKDLNNYNYTPSYNHYNINNQNMmmNLPY**VSGP**STYNAnMI	VSGP	727534450	LOC104724745	Probable WRKY transcription factor 8
	nI**SDGS**KSFLPVDISEESEVSGSDKEDSSWISWFCnLRGNKFLC	SDGS	727423875	LOC104730545	Casein kinase II subunit beta-2-like isoform X1
	QGYV**AGSP**ESSGFHLG	AGSP	727560928	LOC104734746	Protein MEI2-like 4
Antihypertensive (6)	EFCGGTDTKR**VIKP**TEDKRFEEmTnTVGMIEHYMnInHWVC	VIKP	1109068111	LOC104746755	Eukaryotic translation initiation factor 3 subunitC
	GQDHVQPSNMDSPAKKqKTSSqGPDVqIDSGEETLRNPSmE**RPYL**C	RPYL	1109,077495	LOC104750981	Probable inactive tRNA-specific adenosine deaminase-like protein 3
	GQGGqL**LSPY**qGSYNqGQGTPLPGQGQE	LSPY	727647923	LOC104771657	Multiple organellar RNA editing factor 4, mitochondrial-like
	RQGYV**AGSP**ESSGFHLG	AGSP	727560928	LOC104734746	Protein MEI2-like 4
	m**AmQS**QMQLPqFPVMNRSAPQNH	AMQS	727446624	LOC104781817	Transcription factor PIF5 isoform X2
	SDQIRLNFLPqMSDYEAqqHLKMKSDYHQQA**LGYL**PENTNKEMMGLNP	LGYL	727,588,840	LOC104747487	Transcription factor ABORTED MICROSPORES
Anti-cancer (1)	DLLAYERQLAMSKMVGMNPLM**HHPHG**QHALKHAAmGATGSSQGMYDGGFQNA	HHPHG	727573477	LOC104740431	Oligouridylate-binding protein 1B
Anti microbial (2)	GLAGqLHMnSn**KWKW**FSSGDEVLHSGEGPI	KWKW	727605090	LOC104754999	Vacuolar protein sorting-associated protein 41 homolog
	KACCGSGPLRINTCGNRmGPSQ**SYEL**CENVTDYLFFDSSHLTEKAHRqI	SYEL	727617266	LOC104758748	GDSL esterase/lipase 3-like
Anti viral (1)	KACCGSGPLRINTCGNRmGPSQ**SYEL**CENVTDYLFFDSSHLTEKAHRqI	SYEL	727617266	LOC104758748	GDSL esterase/lipase 3-like

In bold the sequence stretches corresponding to potential bioactive activity. Peptide sequence, database sequence, GI number, gene name and protein name are indicated for each peptide. Post-translational modifications are highlighted with lowercase letters: m = oxidated methionine, n = deamidated asparagine, q = deamidated glutamine.

**Table 3:** List of potentially bioactive peptides found in CAMC specimens in DFBP database.

CAMC DFBP					
Bioactivity	Peptides identified	Database SATPdb sequence	Protein origin	Gene	Protein name
Ace inhibitors (13)	EFCGGTDTKR**VIKP**TEDKRFEEmTnTVGMIEHYMnInHWVC	VIKP	1109068111	LOC104746755	Eukaryotic translation initiation factor 3 subunit C
	GA**GAGP**GLGGGIGPDNTLVFFmHDILGGSNP	GAGP	727457383	LOC104786832	Dirigent protein 10-like
	GQDHVQPSNMDSPAKKqKTSSqGPDVqIDSGEETLRNPSmE**RPY**C	RPYL	1109077495	LOC104750981	Probable inactive tRNA-specific adenosine deaminase-like protein 3
	GQGGqLLSPYqGSYNqGQGTPLPGQGQE	YQGS	727647923	LOC104771657	Multiple organellar RNA editing factor 4, mitochondrial-like
	HqSFSYGQESnEFVGSFGASSSYV**AAAT**IG	AAAT	727528607	LOC104722956	Dof zinc finger protein DOF4.3-like
	nGCGDGGGVT**AAAT**NMQEPSIEDK	AAAT	727,470,599	LOC104792737	Patatin-like protein 6
	IKDLNNYNYTPSYNHYNINNQNMmmNLPY**VSGP**STYNAnMI	VSGP	727534450	LOC104724745	Probable WRKY transcription factor 8
	KTFLEqVEILSE**RYRP**DIAEDREDFDNRPYDPED	RYRP	727556889	LOC104732697	RNA polymerase I termination factor-like
	LSLCDnFPqGPGTVVEVVSLVLQRIC**EDLE**A	EDLE	1109046314	LOC104730197	Small subunit processome component 20 homolog
	nI**SDGS**KSFLPVDISEESEVSGSDKEDSSWISWFCnLRGNKFLC	SDGS	727,423,875	LOC104730545	Casein kinase II subunit beta-2-like isoform X1
	RQGYV**AGSP**ESSGFHLG	AGSP	727560928	LOC104734746	Protein MEI2-like 4
	TRESPLTq**GSEN**NmGDSVmNADESV	GSEN	727450232	LOC104783311	DUF724 domain-containing protein 6-like isoform X2
	VSGMNYYACLSMmSL**LIVT**PFAIAVEGPqMWTAGWqNAVSQI	LIVT	727432264	LOC104770334	Phosphate/phosphate translocator 2, chloroplastic-like
Anti-hypertensive (2)	GA**GAGP**GLGGGIGPDNTLVFFmHDILGGSNP	GAGP	727457383	LOC10478683	Dirigent protein 10-like
	GQDHVQPSNMDSPAKKqKTSSqGPDVqIDSGEETLRNPSmE**RPY**C	RPYL	1109077495	LOC104750981	Probable inactive tRNA-specific adenosine deaminase-like protein 3
Anti cancer (1)	SESEEEIRASDDVLAH**DEDDD**E	DEDDD	727425466	LOC104737174	B3 domain-containing protein At5g60130-like
Antithrombotic(6)	MGGPmGmGGPMGNIPAVQGLPAT**GPGG**VPPGYFqGAGSDPMQQQQYM	GPGG	727630269	LOC104764299	Neurogenic protein mastermind-like
	NLGGGPAKNGGKGAPGGGGGGGKG**GPGG**GGENQNQGGGKNGGKnGP	GPGG	727,630,269	LOC104764299	Neurogenic protein mastermind-like
	nnKPmDDFDSP**GPGG**GRGSSSPVSKGQGL	GPGG	727551462	LOC104730762	WPP domain-interacting protein 1-like
	QAVQGLPAm**GPGG**GGGGGASGGAPPGYFqGqVPGS	GPGG	727563725	LOC104735970	S-antigen protein-like
	NAVSQLTnmGPPmPQAPRNMGSGGRFS**GRGDS**GPGHVSSF	GRGDSRGDS	727412897	LOC104776219	Zinc finger CCCH domain-containing protein 36
	TTFmn**CLCR**nGqIDEALKLLGEMKA	CLCR	1109054035	LOC104735908	Pentatricopeptide repeat-containing protein At5g18475-like
DPP IV inhibitory (9)	HqSFSYGQESnEFVGSFGASSSY**VAAAT**IG	VAAA	727528607	LOC104722956	Dof zinc finger protein DOF4.3-like
	VSGMNYYACLSMmSLLIVTPF**AIAV**EGPqMWTAGWqNAVSQI	AIAV	727432264	LOC104770334	Glucose-6-phosphate/phosphate translocator 2, chloroplastic-like
	MGGPmGmGGPMGNIPAVQGLPAT**GPGG**VPPGYFqGAGSDPMQQQQYLAAM	GPGG	727630269	LOC104764299	Neurogenic protein mastermind-like
	NLGGGPAKNGGKGAPGGGGGGGKG**GPGG**GGENQNQGGGKNGGKnGGGP	GPGG	727630269	LOC104764299	Neurogenic protein mastermind-like
	nnKPmDDFDSP**GPGG**GRGSSSPVSKGQGL	GPGG	727551462	LOC104730762	WPP domain-interacting protein 1-like
	QAVQGLPAm**GPGG**GGGGGASGGAPPGYFqGqVPGS	GPGG	727563725	LOC104735970	S-antigen protein-like
	QMLnAHKnGGGGP**GPAG**GK	GPAG	727563725	LOC104735970	S-antigen protein-like
	AKN**GPAG**GRGGGRGGGNGRGRGGn	GPAG	727516553	LOC104718584	Plasminogen activator inhibitor 1 RNA-binding protein-like
	nPVGGILGSQnPGFVQNSM**LPGG**	LPGG	1109025921	LOC104781161	VHS domain-containing protein At3g16270-like
PEP-inhibitory (2)	LAGHAEPVPR**PPPV**PPQLEE	PPPV	1109086438	LOC104755904	Factor of DNA methylation 1-like
	LPEFnNSYTY**LPPV**SGQAMmPVDERPMLYGSNPN	LPPV	727504473	LOC104712991	ETHYLENE INSENSITIVE 3-like 3 protein

In bold the sequence stretches corresponding to potential bioactive activity. Peptide sequence, database sequence, GI number, gene name and protein name are indicated for each peptide. Post-translational modifications are highlighted with lowercase letters: m = oxidated methionine, n = deamidated asparagine, q = deamidated glutamine.

**Table 4:** List of potentially bioactive peptides found in CM specimens in SATPdb and DFBP databases.

CM SATPdb					
Bioactivity	Peptides identified	Database SATPdb sequence	Protein origin	Gene	Protein name
Ace inhibitors (1)	IQEESQQFLnPnE**VVPP**ESnEQQR	VVPP	916445796	atp4	ATPase subunit 4 (mitochondrion)
Antihypertensive (1)	IQEESQQFLnPnE**VVPP**ESnEQQR	VVPP	916445796	atp4	ATPase subunit 4 (mitochondrion)
CM DFBP					
Ace inhibitors (1)	IQEESQQFLnPnE**VVPP**ESnEQQR	VVPP	916445796	atp4	ATPase subunit 4 (mitochondrion)

In bold the sequence stretches corresponding to potential bioactive activity. Peptide sequence, database sequence, GI number, gene name and protein name are indicated for each peptide. Post-translational modifications are highlighted with lowercase letters: m = oxidated methionine, n = deamidated asparagine, q = deamidated glutamine.

## Discussion

The results reported in Table 1 revealed an interesting nutritional profile. The DM content of CAMC, CC and CM partially confirmed what has been reported in the literature by Lolli et al.^14^, Serrapica et al.^15^ Nannucci et al.^17^ and Steppa et al.^18^. It is possible that the differences observed between the same matrices can be attributed to multiple factors, including plant genotype, agrological and meteorological conditions, and harvest type^3^. Furthermore, as reported by Vastolo et al.^3^, great variability between co-products is caused by the processing techniques by which they are obtained. The higher protein content of CAMC is most likely due to the protein value of seeds, the original matrix from which these products are obtained. In fact, as reported earlier, camelina seeds are characterised by a higher protein content than cardoon, which reflects in a greater value in the co-product^10,11^. In general, oil extraction results in the concentration of the protein fraction (30 to 50%), as demonstrated by House et al.^19^ and Ely and Fike^20^ on hemp-based products. This was confirmed for CAMC, however, for CC and CM this did not occur, suggesting that the cardoon seeds used to form the co-products in this study were most probably characterised by a lower protein content than that reported by Genovese et al.^11^ (16.7%).

Nevertheless, as shown in Fig. 1, CC was characterised by a higher protein digestibility than CM, confirming the results of Serrapica et al.^15^. This observation is of fundamental importance as it shows how different oil extraction methods can alter the chemical-nutritional properties of co-products. In fact, as reported by Arrutia et al.^21^, Ancuța and Sonia^22^, the “cake” is the result of the oil extraction directly from the pressing, while the “meal” is obtained by adding another step in the de-oiling process, generally using an organic solvent and high temperatures (> 105 °C), capable of extracting a higher percentage of oil. However, heat treatments, as reported by Teodorowicz et al.^23^, could cause the *Maillard reaction* between amino acids and reducing sugars, which leads not only to a decrease in nutritional value, but also to less digestion by gastric and intestinal enzymes, confirming what was observed in Fig. 1. In parallel, CAMC showed an interesting protein digestibility, even if lower than CC. Although this parameter is not adequately described in the literature, it can be assumed that antinutritional factors such as phytic acid, condensed tannins and erucic acids, present in camelina, are able to decrease protein digestibility by altering the activity of digestive enzymes or forming insoluble complexes with proteins^24^.

Interesting results were also reported for lipid content. CM showed significantly higher content than CC. These results are a direct consequence of the extraction technique adopted, confirming what has been reported by Arrutia et al.^21^. In parallel, higher values observed for CAMC are most probably due to a higher lipid concentration in camelina seed than in cardoon seed, as demonstrated by Genovese et al.^11^, Turco et al.^9^, and Singh et al.^10^. The NDF, ADF, and ADL values observed for CAMC and CM showed differences from those reported by Nannucci et al.^17^ and Singh et al.^10^. Again, as highlighted before, genotype, harvest, agrological and environmental conditions and processing techniques may have influenced the total fibre content^3^. In parallel, CC had a fibre profile highly comparable to that observed by Serrapica et al.^15^. More specifically, the authors reported a content of 46.8, 36.0 and 6.43% for NDF, ADF, and ADL, respectively. Although, as demonstrated by Farinon et al.^25^, adequate fibre consumption may have a functional, mainly prebiotic role at the gastrointestinal level, high levels may affect total digestibility, especially for monogastrics.

Despite this, as shown in Fig. 1, CAMC showed an interesting DM digestibility, confirming the value reported by Moloney et al.^26^ (60.0 ± 3.01%). This result is highly comparable to the DM digestibility of soy protein extract, one of the main feed matrices used in the livestock sector, as demonstrated in our previous work^27^. At the same time, different methods for oil extraction observed differences in DM digestibility between CC and CM, suggesting that the use of high temperatures combined with solvents could affect the nutritional aspect of the food/feed matrix. In light of the above, CAMC, CC, CM are characterised by an interesting nutritional profile and digestibility, although the high level of fibre suggests a greater use for ruminants. Nevertheless, Zumbo et al.^28^ and Buccioni et al.^29^ investigated their use in poultry and pig sector. As reported by the authors, although no difference in growth performance was found, the acid profile of meat and eggs was improved, suggesting a potential use in monogastric farming as an alternative to soy-protein. Specifically in this study, the results obtained by CAMC and CC were higher than those of CM, identifying cake as a preferred product compared to meal. This data enhances the use of pressed cake as a protein-rich ingredient in animal feed, which is regulated by the Commission Directive 2008/76/EC^30^.

In parallel, as reported in Fig. 2, for TPC, no statistically significant difference was observed between the samples at the end of the oral phase. These results are related to the behaviour of phenolic compounds during the digestive process. More specifically, the oral phase, as reported by Hur et al.^31^, due to the short contact period between saliva and the food matrix, does not affect the availability of phenolic compounds in this stage, resulting in no high values in the TPC. The increase in TPC following filtration is related to the changes undergone by phenols during the digestive process. In fact, during the gastric phase, the acidic pH leads to the breaking of protein chains and polysaccharide compounds, mainly fibres, causing the release of phenolic compounds^32,33^. Later, although at the intestinal level these values tend to decrease, as the alkaline pH and the action of pancreatin transform phenols into compounds with different structures and bioactivity^34^, as reported by Tarko et al.^35^, about 48% of phenolic compounds are digested in the small intestine, and only a small fraction (about 10%) remains bound to the food matrix, explaining the results observed for Fig. 2. Considering individual matrices, as reported by Terpinc et al.^36^, CAMC is characterised by the presence of several phenolic compounds (rutin, catechin, quercetin, quercetin-3-O-glucoside, protocatechuic acid, p-hydroxybenzoic acid, ellagic acid, sinapic acid, salicylic acid and 4-vinyl derivatives of hydroxycinnamic acids), found to be equal to or greater than those contained in the seed, suggesting that oil extraction results in their concentration in the cake. However, TPC following in vitro digestion is not properly described in the literature. Despite this, it is presumable that the values observed in Fig. 2 are influenced by the presence of non-starch polysaccharides, compounds that can increase intestinal viscosity (a situation also observed in this experiment), thus trapping phenols and preventing their correct detection^10,37^. At the same time, the cardoon seed, as reported by Piluzza et al.^38^, is also characterised by the high presence of phenolic compounds, which are most probably preserved and concentrated during the production of the cake, as previously reported for CAMC. Although, as reported by Juániz et al.^39^, only a few phenolic compounds of CC were observed following in vitro digestion, this is not confirmed in this study. Most probably, as reported by Petropoulos et al.^12^, genotypic differences and cultivation conditions may play an important role in the profile of phenolic compounds and could justify the conflicting results of the different studies. Finally, the significant differences observed between CC and CM are most probably due to the high temperatures adopted to obtain CM, able to cause a reduction in TPC, as reported by Ghafoor et al.^40^.

As shown in Fig. 3, FRAP and ABTS assays are characterised by the same trend of TPC, suggesting that phenolic compounds are actively involved in the determination of antioxidant activity, confirming what was described by Wojtunik-Kulesza et al.^34^. Again, in addition to the food matrix, it is crucial to consider the conditions of digestion. The limited duration of the oral phase (2 min) results in a brief interaction between the oral enzymes and the food bolus, without major changes in phenolic compounds and consequently in relative antioxidant activity^31^. However, as reported by Ginsburg et al.^41^, the saliva, but more specifically salivary albumin, mucins and proline-rich proteins, enable the solubilisation of phenols by increasing their availability, digestibility, absorption and antioxidant activity in subsequent digestion steps. This would explain the higher values obtained following filtration at the end of digestion. At the same time, the increase in antioxidant activity, as reported by Wojtunik-Kulesza et al.^34^, is the result of the action of the acidic pH at stomach level, which increases the antioxidant power of phenolic compounds by mainly strengthening their ability to donate electrons, the principle of action of the ABTS assay. The latter plays a key role as it explains why higher values were recorded for ABTS than FRAP.

In parallel, as reported by Ngo et al.^42^, one of the major forms of oxidative stress is caused by hydroxyl radicals, as they are capable of interacting involving molecules such as DNA, proteins, lipid membranes and amino acids, consequently causing multiple cellular damages. For this reason, antioxidant activity has also been assayed using the ORAC method, an assay that quantifies compounds capable of breaking peroxyl radical chains^43^. However, as shown in Fig. 3, ORAC reported an opposite trend to that observed for ABTS and FRAP. Although several studies have shown a positive correlation between TPC and ORAC in various fruits and vegetables, a high TPC does not always correspond to a high ORAC^44^. In parallel, these discrepancies can also be observed between antioxidant assays, especially when the samples analysed contain a high profile of phenolic compounds and antioxidant molecules with different mechanisms of action, as demonstrated by Zhou et al.^44^. This difference could also occur in different species of the same plant, as reported by Gutiérrez-Grijalva et al.^45^. More precisely, the authors observed how in three different species of oregano (*Hedeoma patens, Lippia graveolens, Lippia palmeri*), although antioxidant activity increased between the oral and gastric phases with ABTS, with the ORAC assay, the trend was the opposite, suggesting how plant matrices are characterised by multiple antioxidant compounds that can operate independently and differently^45^. Furthermore, as previously reported, during the digestive process, phenolic compounds, but in general antioxidant compounds, undergo profound changes. In this case, as shown in Fig. 3, molecules with peroxyl chain-breaking activity appear to remain stable during the oral phase and are degraded at the end of digestion.

In spite of this, CAMC, CC and CM proved to be co-products characterised by a phenolic profile and antioxidant activity comparable or superior to that of soy protein extract (1841.11 ± 23.02 mg TAE/100 g; 2968.49 ± 93.87 mg TE/100 g; 48.14 ± 16.40 mg FeSO_4_/100 g, for TPC, ABTS and FRAP, respectively)^27^, suggesting their possible use as functional substitute matrices.

As reported by Ahmad et al.^46^, the study of ACE inhibitory activity in plant matrices plays a key role in the food/feed sector, although this has not yet been fully investigated.

No detectable values were reported by the assay for CAMC and CC at the beginning of the oral phase, while increasing level were observed after in vitro digestion, confirming the findings of Vermeirssen et al.^47^. The authors reported how the in vitro digestive process of plant and animal proteins increased their ACE inhibitory activity. Most probably, this is the result of the action of enzymatic proteolysis during the digestion, which results in the release of bioactive peptides^48^. At the same time, the CC showed an interesting trend, with values already present at the end of the oral phase (18.03 ± 0.33%), partly confirming what Akillioglu and Karakaya described^48^. Indeed, the authors reported how ACE inhibitory peptides may be present in intact dietary proteins.

As stated above, the search for potentially bioactive peptides was performed on all CAMC, CC and CM sample datasets using the Structurally Annotated Therapeutic Peptides database SATPdb^49^ and the Food-derived bioactive peptides database, DFBP^50^.

The main activity is related to ACE inhibitor and antioxidant activity, in accordance with previous reports^51,52^. Oxidation is an important contributor to many human and animal diseases, including those cardiovascular that are associated with generation of increased amount of reactive oxygen species (ROS)^53^. Therefore, the protection against free radicals induced by oxidation is a key factor in preventing such diseases^54^. An important role in the regulation of blood pressure is played by ACE, that catalyses the hydrolysis of the inactive decapeptide angiotensin I to the potent vasoconstrictor angiotensin II^55^. For this reason, ACE inhibitors with antioxidant activity are widely used in hypertension treatments^53^. For more than ten years, there has been some evidence that vegetables proteins and their hydrolysates are potentially excellent sources of antioxidants and antihypertensive peptides^56^. In particular, proteins isolated from flax seed^57^, rapeseed^58^, pumpkin oil cake^59^ and wheat gluten^60^ have been reported to show both ACE inhibitory and antioxidant activities. In keeping, the peptidomics results identified three peptides that contain a sequence stretch corresponding to ACE inhibitors activity (Tables 2, 3), in CAMC. They originated from the dirigent protein 10-like, patatin-like protein 6 and WRKY transcription factor (TF) 8, respectively, whose involvement in oxidative stress response has already been described in literature.

The dirigent protein 10-like is involved in the synthesis of lignin that plays pivotal roles in plant defence responses, both against biotic and abiotic stresses, exerting a variety of functions such as antimicrobial, antiviral, antioxidant and anti-cytotoxic^60^. Patatin is the trivial name given to a family of glycoproteins that make up > 40% of the total soluble protein in potato (*Solanum tuberosum*) tubers that serves as storage protein, which globular structure offers versatile bioactive sites for numerous bifunctionalities, such as antioxidant^61^. A previous study conducted by Fu et al.^62^ proved that patatin could capture radical in a concentration-dependent manner exhibited antioxidant activities.

The probable WRKY TF 8 belongs to one of the major plant protein superfamilies that plays an important role in the regulation of transcriptional reprogramming associated with plants stress responses^63^. Recent studies have shown that WRKY-TF is induces through ROS and contributed to the ROS elimination transformation pathway^64^.

Also plasminogen activator inhibitor 1 RNA-binding protein like generates a peptide involved in oxidative stress response, in addition to dipeptidyl-peptidase IV (DPP IV)-inhibitory activity (Tables 2, 3). DPP-IV plays an essential role in glucose metabolism. In this context, food-protein-derived DPP-IV inhibitors are promising glycemic regulators which may act by preventing the onset of type 2 diabetes and the related oxidative stress^65^.

The peptidomics results on camelina are, therefore, well in accordance with the characterisation of its antioxidant profile described above. The same can be described for CM specimen. In this case the analysis of bioactive peptides identified one peptide, exclusively present in CM samples, containing a sequence stretch corresponding to one peptide with ACE inhibitors and antihypertensive potential activity (Table 4).

Altogether, peptidomic results show the presence of several bioactive peptides with ACE inhibitors and antioxidant activities, according with the characterization of the antioxidant profile. These results, combined with the high content of phenolic compounds, perfectly explain the significant antioxidant activity measured in camelina and cardoon samples.

In conclusion, CAMC, CC and CM showed an interesting nutritional profile and high digestibility. In parallel, CAMC, CC and CM revealed a high antioxidant profile, superior to that of soy protein extract, a matrix highly used in the feed industry. The antioxidant capacity and ACE inhibitory activity seem to be positively influenced by presence of phenols and bioactive peptides. Although, the samples analysed showed relevant functional profile, the high fibre content, as well as, the possible presence of anti-nutritional factors and the high variability between co-products batches need to be considered for their application in the feed sector. These insights, coupled with future in vivo trials, will be crucial to evaluate the efficacy on the health and performance of farm animals.

## Methods

### Nutritional profile determination

The determination of nutritional profile of commercially available CAMC (Panghea s.p.a., Milan, Italy), CC and CM (Novamont s.p.a., Novara, Italy) was performed following the official methods of analysis according to AOAC^66^. Specifically, DM was obtained by drying the samples at 65° C for 24 h (AOAC method 942.05). Ashes were determined by incinerating the samples at 550° C for 3 h (AOAC method 942.05). Crude protein content was assessed by the Kjeldahl method (AOAC method 2001.11), and ether extract by ether extraction with the Soxtec system (DM 21/12/1998). Finally, the fibrous fractions were identified using the protocol developed by Van Sost et al.^67^. The procedure described above was performed at least in triplicate.

### In vitro digestion and digestibility

The in vitro digestion protocol was performed according to Regmi et al.^68^. Aliquots (1 mL), corresponding to the soluble fraction, were taken at the beginning (oral phase) and at the end of digestive process (following filtration with 10 kDa filters), and immediately frozen at −20° C to measure TPC and antioxidant activity.

At the end of digestion, different replicates were vacuum filtered using a porcelain funnel covered with filter paper (Whatman 54, Florham Park, NJ). The undigested fraction (UF) was subsequently dried overnight at 65° C and quantified to measure DM digestibility, using the following formula:

$${\text{Digestibility (\% DM) = (Sample DM }}{-}{\text{UF DM)/sample (DM) }} \times { 100 }$$

Subsequently, protein digestibility was also measured. Specifically, UF on the filters was analysed according to the Kjeldahl method for CP content quantification (AOAC method 2001.11).

The procedures described above were analysed in biological triplicate (n = 3). For each biological replicate, the technical duplicate was considered.

### Total phenolic content

Reagents (tannic acid, methanol, Folin-Ciocalteu reagent (FC) and sodium carbonate (Na_2_CO_3_) were purchased from Sigma Chemical Co. (St. Louis, MO, USA).

The TPC was determined following the protocol of Attard^69^ with minor modifications^33^. More precisely, tannic acid was prepared in five (1:2) dilutions in distilled water (0 to 480 µg/mL). FC reagent was diluted (1:10) with distilled water, while Na_2_CO_3_ was prepared at a concentration of 1 M. Subsequently, 100 µL of sample was incubated for 20 min in the dark at room temperature (RT) with 500 µL of FC and 400 µL of Na_2_CO_3_. At the end of the incubation, the samples were read using a spectrophotometer at a wavelength of 630 nm. Each analysis was performed including appropriate solvent blanks. Data were expressed in terms of tannic acid equivalent (mg TAE/100 g).

Total phenolic content was analysed for each replicate of the digestion. For each biological replicate, at least the technical duplicate was considered.

### ABTS assay

Reagents (ABTS, 6-hydroxy-2,5,7,8-tetramethochroman-2-carboxylic acid (Trolox), and potassium persulphate (K_2_S_2_O_8_)) were purchased from Sigma Chemical Co. (St. Louis, MO, USA).

The ABTS method was tested following the protocol of Re et al.^70^. Specifically, Trolox (2.5 M) was used as the antioxidant standard and prepared in six dilutions (0 to 2000 µM/mL). Subsequently, 88 µL of K_2_S_2_O_8_ (2.45 mM) was added to 5 mL of ABTS (7 mM) to form the ABTS radical cation (ABTS^+^) and incubated for 16 h in the dark at RT before use. For the quantification of antioxidant activity, the ABTS^+^ solution was diluted with ethanol to an absorbance value of 0.7 ± 0.02 at 734 nm. Then, 10 µL of sample was added to 1.0 mL of diluted ABTS^+^ solution (0.7 ± 0.02) and incubated at RT in the dark for 6 min. At the end of the incubation, the samples were read using a spectrophotometer at a wavelength of 734 nm. Each analysis was performed including appropriate solvent blanks. Data were expressed in terms of Trolox equivalent (mg TE/100 g).

The ABTS assay was analysed for each replicate of the digestion. For each biological replicate, at least the technical duplicate was considered.

### FRAP assay

The reagents (ferric chloride hexahydrate [FeCl_3_ (6 H_2_O)], ferrous sulphate (FeSO_4_), sodium acetate trihydrate, glacial acetic acid, and 2, 4, 6-tripyridyl-s- triazine (TPTZ)) were purchased from Sigma Chemical Co. (St. Louis, MO, USA). The FRAP assay was performed following the protocol of Lanzoni et al.^33^. The reagents were constituted as follows. a) Buffer acetate (300 mM, pH 3.6): 2.69 g sodium acetate trihydrate were dissolved in 16 ml glacial acetic acid and diluted to 1 L with distilled water; b) TPTZ solution (10 mM): 31.2 mg of TPTZ were dissolved in 10 mL of HCl (40 mM); c) [FeCl_3_ (6H_2_O)] solution (20 mM): 0.054 g of [FeCl_3_ (6H_2_O)] were dissolved in 10 mL of distilled water. While, FeSO_4_ was used as an antioxidant standard and prepared in six dilutions from 0 µM/L to 1500 µM/L. Subsequently, the working solution (FRAP reagent) was prepared by combining 2.5 mL of TPTZ solution + 2.5 mL of [FeCl_3_ (6H_2_O)] solution + 25 mL of acetate buffer. For quantification of antioxidant activity, 10 µL of each sample was added to 300 µL of FRAP reagent and incubated at RT for 20 min in the dark and read at 595 nm. Each analysis was performed including appropriate solvent blanks. Data were expressed in mg FeSO_4_/100 g. FRAP was analysed for each replicate of the digestion. For each biological replicate, at least the technical triplicate was considered.

### ORAC assay

Antioxidant capacity was determined using the ORAC kit (ab233473, Abcam, Cambridge, MA, USA). Fluorescein was used as substrate for the reaction, while Trolox (0.2 mM) was used as standard for calibration curve (0 to 50 µM/mL). More precisely, 25 µL of digested sample were incubated with 150 µL of fluorescein in a 96-well plate and incubated at 37° C for 30 min. At the end of the incubation, 25 µL of Free Radical Initiator solution was added to each well to complete the reaction. Fluorescence was measured every 5 min for one h at 37° C at Ex/Em = 480/520 nm. All determinations were performed at least in triplicate. Each analysis was performed including appropriate solvent blanks. Data were expressed in mg TE/100 g.

The ORAC assay was analysed for each replicate of the digestion. For each biological replicate, at least the technical duplicate was considered.

### ACE inhibition assay

The ACE inhibition assay was performed as reported by Giromini et al.^71^ with minor modifications^72^, using furanacroloyl-Phe-Glu-Glu (FAPGG) as a synthetic substrate for the ACE-1 enzyme. More specifically, 150 µL of FAPGG was incubated at 37° C for one min. At the end of the incubation, 10 µL of each sample and 10 µL of ACE-1 (15 mU) were added to the substrate, thus initiating the kinetic reaction. The kinetic reaction was measured with Synergy KTX at a wavelength of 340 nm for each minute (30 min in total), using captopryl as a positive control. Hydrolysis of FAPGG by the enzyme ACE-1 caused a decrease in absorbance at 340 nm. A 100% ACE-1 activity would indicate complete inhibition of the enzyme.

$$\% {\text{ACE}} - 1{\text{ inhibition }} = \, \left( {\left( {{\text{Absno sample}} - {\text{Abssample}}} \right)/{\text{Absno sample}}} \right) \times 100$$

Absno sample is the absorbance of the enzyme-substrate mixture in the absence of sample, while Abssample is the absorbance of the enzyme-substrate mixture in the presence of the matrices under study. The experiment was performed at least in duplicate.

### MS/MS peptidomic methods

To identify endogenous peptides, the permeate samples were analysed by peptidomic strategy by LC-nano ESI tandem mass spectroscopy, using a shotgun-label free approach, without any digestion prior to MS/MS^73,74^.

Following the digestion process, the samples were initially filtered using paper filters (Whatman 54, Florham Park, NJ), as previously reported^68^. Subsequently, the filtered component, corresponding to the digested fraction, was ultra-filtered using centrifuge filters (10 kDa) (Pierce™, Protein Concentrator PES, Thermo Fisher Scientific, UK), for high weight protein depletion^75^. Finally, the obtained fraction was lyophilised prior to mass spectrometric (MS) analysis.

More precisely, before MS, freeze-dried supernatant of CAMC, CC, CM, containing peptides and low molecular weight proteins, was dissolved in 0.3% (v/v) formic acid and desalted (Zip-Tip C18, Millipore, Billerica, MA, USA)^74,75^. Nano-HPLC coupled to MS/MS analysis was performed on Dionex Ultimate 3000 HPLC system with an EASY-SprayTM 2 µm 15 cm × 150 µm capillary column filled with 2 µm C18 100 Å particles, connected to a Q-Exactive Orbitrap (Thermo Fisher Scientific, San Jose, CA, USA) using mobile phase A (0.1% formic acid in water) and mobile phase B (0.1% formic acid in acetonitrile 20/80, v/v) at a flow rate of 0.300 µL/min. The temperature was set to 35ºC.

The acquired raw files were subjected to data analysis using Proteome Discoverer software (version 1.4). The searches were performed against the NCBI reference *Carduus* proteome (updated on November 2023; 867 sequences) for CC and CM samples, and against the NCBI reference *Camelina* proteome (updated on November 2023; 116195 sequences) for CAMC. The enzyme specificity was set as unspecific and methionine oxidation, and asparagine/glutamine deamidation were set as variable modifications^75,76^. Only peptides with high confidence were included for positive identification.

All peptides were searched in SATPdb^49^, a database of structurally annotated therapeutic peptides, and in DFBP, a database of food-derived bioactive peptides^50^. To consider possible further proteolysis, the search was performed keeping a minimum sequence length of four amino acids and applying an “IF” nested function to a matrix which compared the sequence of each peptide found with the ones of the database (Micrososft Excel 2023, version 16.80).

The mass spectrometry raw data have been deposited in the ProteomeXchange Consortium (http://proteomecentral.proteomexchange.org) via the PRiDE partner depository with the dataset identifier PXD049333.

### Statistical analysis

Chemical analysis, DM and protein digestibility were analysed using one-way Anova followed by Tukey’s multiple comparison test. TPC and antioxidant activities following in vitro digestion were analysed by two-way Anova (*Time x Treatment*) followed by Tukey’s multiple comparison test, using GraphPad Prism 9 9.3.1 (GraphPad Software Inc., San Diego, CA, USA). All data are reported as mean ± standard error of the mean (SEM) of at least three independent experiments. Values are considered statistically significant for a 95% confidence interval (*p*-value = 0.05).

### Supplementary Information

Supplementary Legends.

Supplementary Table S1.

Supplementary Table S2.

## Data Availability

The mass spectrometry peptidomic data have been deposited in the PRIDE partner depository for the ProteomeXchange Consortium with dataset identifiers: PXD049333. The account details and the other data obtained in this work (digestibility, total phenolic content and antioxidant activity after in vitro digestion) are available from the corresponding author on reasonable request.
