# Supplementary material for: Antioxidant capacity and peptidomic analysis of in vitro digested Camelina sativa L. Crantz and Cynara cardunculus co-products
Source: Sci Rep. 2024 Jun 24;14:14456. doi: 10.1038/s41598-024-64989-3 (PMC11196266; doi:10.1038/s41598-024-64989-3)
Supplement: Supplementary file 1 — Supplementary Legends. [file 41598_2024_64989_MOESM1_ESM.docx]

**Supplementary materials**

**Supplementary Table S1:** Peptides identified in Camelina cake (CAMC). Peptide sequence, modifications, MH+ [Da], peptide length, GI number, gene name and protein name are indicated for each peptide. Post-translational modifications are highlighted with lowercase letters: m=oxidated methionine, n= deamidated asparagine, q=deamidated glutamine. Unique peptides: X indicates peptides exclusively present in the specific protein.

**Supplementary Table S2:** Peptides identified in Cardoon cake (CC) and Cardoon meal (CM). Peptide sequence, modifications, MH+ [Da], peptide length, GI number, gene name and protein name are indicated for each peptide. Post-translational modifications are highlighted with lowercase letters: m=oxidated methionine, n= deamidated asparagine, q=deamidated glutamine. Unique peptides: X indicates peptides exclusively present in the specific protein. Peptides commonly identified in CC and in CM are in bold.
